# Layer-by-Layer Organic Photovoltaic Solar Cells Using a Solution-Processed Silicon Phthalocyanine Non-Fullerene Acceptor

**DOI:** 10.1021/acsomega.1c05715

**Published:** 2022-02-22

**Authors:** Marie D. M. Faure, Chloé Dindault, Nicole A. Rice, Benoît H. Lessard

**Affiliations:** † Department of Chemical and Biological Engineering, University of Ottawa, 161 Louis Pasteur, Ottawa, Ontario, Canada K1N 6N5; ‡ School of Electrical Engineering and Computer Science, University of Ottawa, 800 King Edward Ave., Ottawa, Ontario, Canada K1N 6N5

## Abstract

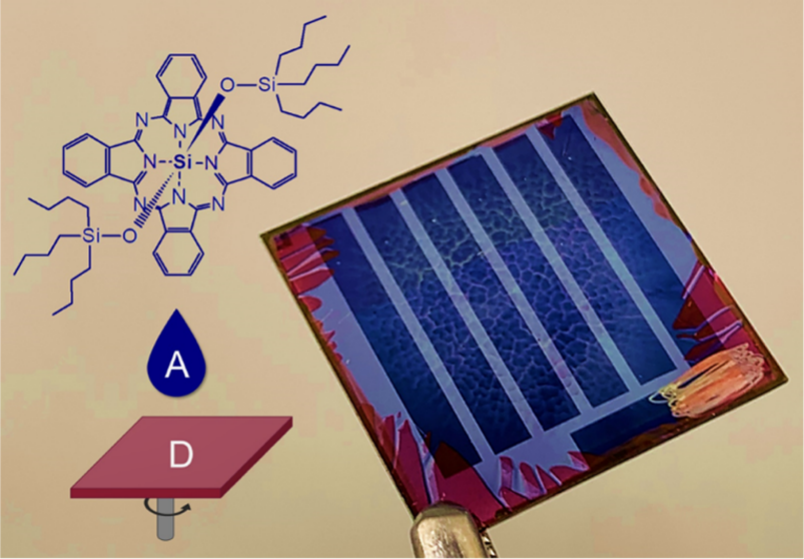

Silicon phthalocyanines (SiPcs) are promising, inexpensive, and easy to synthesize non-fullerene acceptor (NFA) candidates for all-solution sequentially processed layer-by-layer (LbL) organic photovoltaic (OPV) devices. Here, we report the use of bis(tri-*n*-butylsilyl oxide) SiPc ((3BS)_2_-SiPc) paired with poly(3-hexylthiophene) (P3HT) and poly[(2,6-(4,8-bis(5-(2-ethylhexyl)thiophen-2-yl)-benzo[1,2-b:4,5-b′]dithiophene))-*alt*-(5,5-(1′,3′-di-2-thienyl-5′,7′-bis(2-ethylhexyl)benzo[1′,2′-c:4′,5′-c′]dithiophene-4,8-dione))] (PBDB-T) donors in an LbL OPV structure. Using a direct architecture, P3HT/(3BS)_2_-SiPc LbL devices show power conversion efficiencies (PCEs) up to 3.0%, which is comparable or better than the corresponding bulk heterojunction (BHJ) devices with either (3BS)_2_-SiPc or PC_61_BM. PBDB-T/(3BS)_2_-SiPc LbL devices resulted in PCEs up to 3.3%, with an impressive open-circuit voltage (*V*_oc_) as high as 1.06 V, which is among the highest *V*_oc_ obtained employing the LbL approach. We also compared devices incorporating vanadium oxide (VOx) or poly(3,4-ethylenedioxythiophene) polystyrene sulfonate (PEDOT:PSS) as a hole transporting layer and found that VOx modified the donor layer morphology and led to improved *V*_oc_. Probing the composition as a function of film layer depths revealed a similar distribution of active material for both BHJ and LbL structures when using (3BS)_2_-SiPc as an NFA. These findings suggest that (3BS)_2_-SiPc is a promising NFA that can be processed using the LbL technique, an inherently easier fabrication methodology for large-area production of OPVs.

## Introduction

Organic photovoltaics (OPVs) are capable of rivaling the performance of other solar technologies, with state-of-the-art OPV devices exhibiting power conversion efficiencies (PCEs) as high as 18%.^1−3^ This improved efficiency, combined with the potential of semitransparency, flexibility, and low-cost mass production through techniques such as roll-to-roll printing, has been the main reason for continuing research interest.^4,5^ However, for OPVs to become competitive, the selection of active materials, their synthetic complexity, as well as the processes to fabricate and assemble the different layers, is critical. Bulk heterojunction (BHJ) morphology has often been preferred over planar heterojunction (PHJ) morphology, due to significant improvements in device performance.^6−9^ Compared to the PHJ, which has a defined interface between the independently deposited donor and acceptor materials and therefore limited active area available for charge dissociation, the bulk blend of the acceptor and donor materials in BHJ results in the increased interfacial area.^10,11^ However, the random mixing of materials in BHJs makes it challenging to consistently reproduce device performances, particularly high performance with larger area devices, and complicates isolation of the photocharge behavior as a result of morphology changes, all of which significantly hinder a straightforward transition from lab-scale fabrication to mass production.^12,13^

A pseudo-bilayer configuration provides a convenient alternative, where the sequential layer-by-layer (LbL) deposition does not hinder favorable intermixing of the active layers while simultaneously providing a fabrication technique that is easier to optimize and translate to commercial printing processes.^14^ In LbL, the first active layer is often deposited via solution deposition, followed by either thermal evaporation^15,16^ or, more commonly, solution deposition of the second layer.^17−23^ All-solution LbL deposition can promote a more efficient morphology, with a vertical phase separation resulting in increased donor and acceptor concentrations at the respective electrodes and an intermixed region between the electrodes (fuzzy interface).^24,25^ This composition provides enough interfacial contact for excitons to be dissociated, with free charges readily extracted into neat layers to reduce unwanted charge recombination. Additionally, donor swelling and regional depth can be easily tuned and controlled through parameters such as solvents,^26−28^ additives,^29,30^ or thermal treatments.^25,31,32^ Compared to BHJ devices, LbL devices have demonstrated better mechanical and thermal stability and are more robust to variances in experimental parameters including increased surface area^22,23,33^ while achieving equivalent or superior performances for many donor/acceptor systems. Efficiencies as high as 13% have been reported for spin-coated devices,^34^ over 16% for small-area blade-coated devices, and an OPV record of 12% for a large-area blade-coated LbL devices of 11.82 cm^2^.^23^

Preeminent BHJ and LbL devices are normally achieved with novel small-molecule non-fullerene acceptors (NFAs) based on fused acceptor–donor–acceptor push–pull architectures,^20−23,34−36^ such as (3,9-bis(2-methylene-(3-(1,1-dicyanomethylene)-indanone))-5,5,11,11-tetrakis-(4-hexylphenyl)dithieno[2,3-d:20,30-d0]-s-indaceno[1,2-b:5,6-b0]dithiophene) (ITIC).^22,34^ While these elaborate architectures enable favorable molecule properties in OPVs, they require multiple complex synthetic steps with very low (<1%) overall synthetic yields, prohibiting commercialization of this technology^37^ and emphasizing the need for simple, inexpensive, and high-performing OPV materials that can be synthesized and purified through scalable processes.

**Figure 1:**
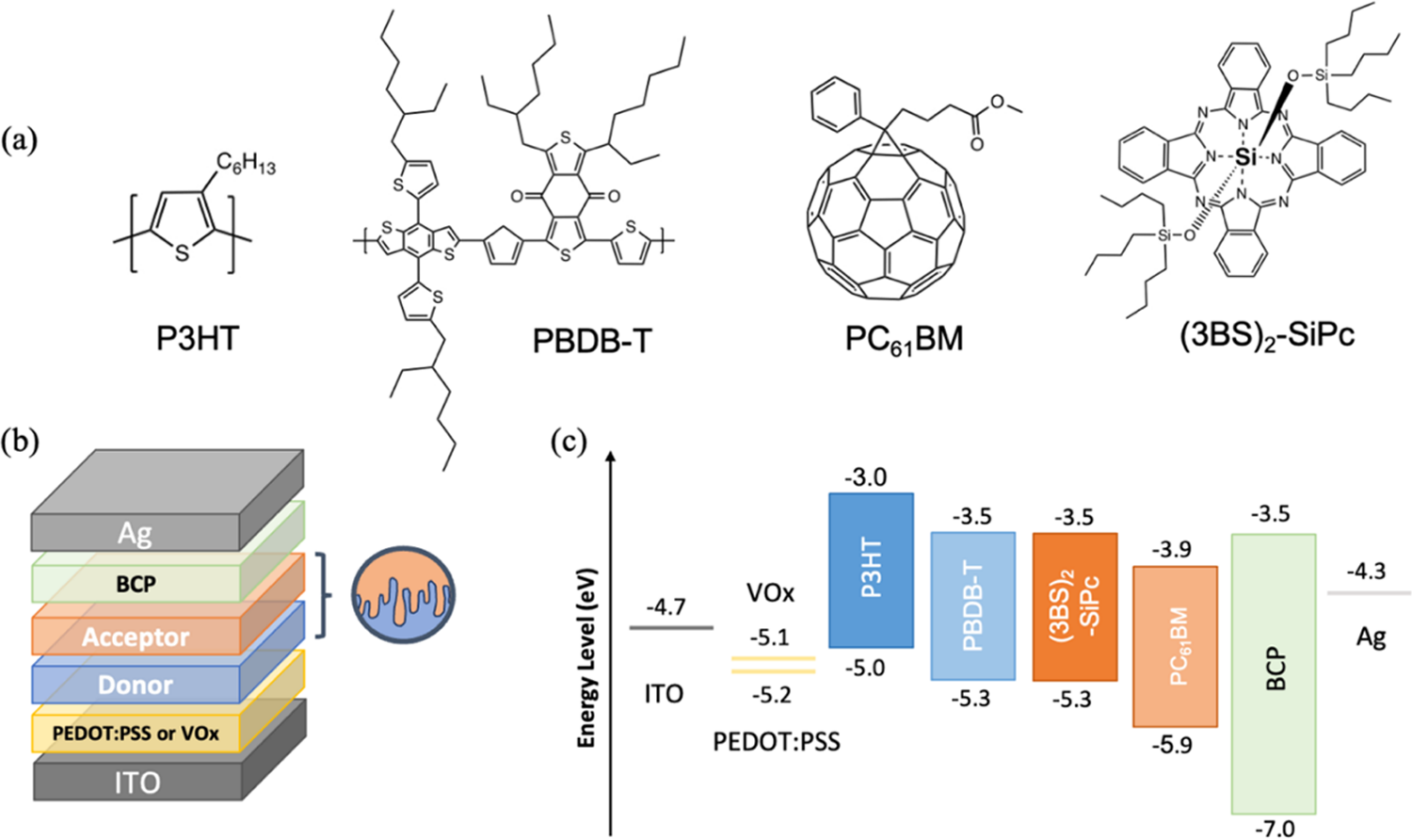
(a) Molecular structures of materials used in the active layer, (b) layer-by-layer direct device structure, and (c) electronic energy levels for all materials incorporated into OPVs.

Silicon phthalocyanines ((R)_2_-SiPc) are established molecules in the dye and pigment industries due to their chemical stability and low production costs.^38^ (R)_2_-SiPcs are tetravalent molecules that can undergo simple axial functionalization through straightforward and scalable chemistry,^39,40^ providing a synthetic handle for improving solubility and tuning aggregation in the solid state,^41^ while modification of the (R)_2_-SiPc macrocycle can be used to adjust the frontier energy levels.^42^ (R)_2_-SiPc derivatives have been investigated in BHJ devices, as either ternary additives or NFAs. An addition of 3 wt % (R)_2_-SiPc derivative as a ternary additive in a poly(3-hexylthiophene):phenyl-C_61_-butyric acid methyl ester (P3HT:PC_61_BM) blend improved photocurrent generation and increased the PCE by 25% through enhanced light absorption around 700 nm.^43^ When bis(tri-*n*-butylsilyl oxide) SiPc ((3BS)_2_-SiPc, Figure 1) was used as an NFA with P3HT or poly[(2,6-(4,8-bis(5-(2-ethylhexyl)thiophen-2-yl)-benzo[1,2-b:4,5-b′]dithiophene))-*alt*-(5,5-(1′,3′-di-2-thienyl-5′,7′-bis(2-ethylhexyl)benzo[1′,2′-c:4′,5′-c′]dithiophene-4,8-dione))] (PBDB-T), BHJ OPVs with an averaged *PCE* of 3.6 and 3.4% were obtained, respectively, with *V*_oc_ surpassing 1 V for the devices with PBDB-T.^44^ Moreover, under reduced illumination, (3BS)_2_-SiPc devices retained higher PCE compared to fullerene-based devices, affording exciting opportunities for indoor applications.^44^ Insoluble phenoxylated SiPc derivatives have been incorporated into bilayer devices through evaporation,^40,45,46^ and Bender and co-workers recently reported the use of a boron subphthalocyanine as an NFA in all-solution-processed LbL OPV devices with PCE up to 3.6%.^47^ To the best of our knowledge, all-solution-processed LbL OPV devices using soluble (R)_2_-SiPc derivatives have never been reported.

The majority of reported LbL-fabricated OPV devices utilize a direct structure incorporating poly(3,4-ethylenedioxythiophene) polystyrene sulfonate (PEDOT:PSS) as the hole transporting layer (HTL).^14^ PEDOT:PSS is a highly conductive (between 2 × 10^–3^ and 1 S.cm^–1^) transparent (>75% in the visible range) polymer, normally processed in aqueous solvents to facilitate orthogonal processing.^48,49^ However, PEDOT:PSS is acidic (pH ≈ 1–4),^48^ degrading both its interface to the ITO electrode and the organic active material, causing indium diffusion into the PEDOT:PSS layer and shortening device lifetime, respectively.^50,51^ Since the early 2000s, transition-metal oxides (TMO) with high work functions, such as MoO_3_, WO_3_, NiO, ZnO, and V_2_O_5_, have attracted interest as charge-transporting layers in organic electronic devices.^52^ TMOs in OPVs can avoid several of the aforementioned pitfalls with PEDOT:PSS, increasing both efficiency and stability of the devices.^53,54^ While most TMOs are thermally evaporated, vanadium oxide (VOx) can be easily obtained from a soluble vanadium precursor (like vanadyl acetylacetonate or vanadium oxytriisopropoxide) dissolved in isopropanol and deposited in ambient conditions without any post-treatment to yield amorphous, smooth layers with electrical properties comparable to those of evaporated V_2_O_5_,^54,55^ making it a promising candidate for the replacement of PEDOT:PSS in fully solution-processed OPVs.

In this study, we employed (3BS)_2_-SiPc as an NFA in all-solution-processed LbL devices and compared device performance to both analogous and P3HT:PC_61_BM BHJ devices. We paired it with P3HT, which remains one of the most commercially viable polymers for OPVs despite declining interest from academia,^56,57^ and with PBDB-T, a high-performing p-type conjugated polymer. We also explored the use of VOx as an alternative to PEDOT:PSS as the HTL. We demonstrate that devices fabricated by the LbL approach perform as well as, and sometimes outperform, analogous BHJ devices, with (R)_2_-SiPc-based LbL devices characterized to have PCEs of ≈3% and *V*_oc_ > 1 V, which are among the highest *V*_oc_ reported for OPVs fabricated through the LbL approach.

## Results and Discussion

(3BS)_2_-SiPc (Figure 1a) is a promising candidate as an acceptor molecule for OPVs due to its high solubility, proven performance as an acceptor in BHJ OPVs,^41,44,58^ and its elevated n-type mobility as reported in organic thin-film transistors.^59,60^ However, its performance as an NFA in LbL OPV devices has yet to be evaluated. In the previous study, BHJ cells were fabricated with an inverted structure (glass/ITO/ZnO/active layer/MoOx/Ag).^44^ Inverted LbL structures are rare in the literature compared to direct LbL devices, due to the convenience of first depositing the donor polymer followed by deposition of a small-molecule acceptor.^14^ For a more accurate comparison between our BHJ and LbL devices, both were fabricated with a direct (glass/ITO/HTL/active layer/BCP/Ag, Figure 1b) architecture, using either PEDOT:PSS or VOx as the hole transporting layer. Additionally, baseline P3HT:PC_61_BM devices were also prepared.^7^

While LbL device structures offer many advantages over BHJ, significant initial optimization of processing conditions is required for new systems. For example, solvent combinations (immiscible or miscible), dispensing volumes, dispensing kinetics, spin rates, and thermal treatments can all play significant roles in the resulting film morphology and device performance. Details of the full optimization of LbL devices prepared in this study can be found in the Supporting Information (Tables S1 and S2). We found the solvent combination that yielded optimal device performance was chloroform (CF) for the donor polymer (P3HT) and chlorobenzene (CB) for the acceptor molecule ((3BS)_2_-SiPc). The low boiling point of CF facilitates the rapid formation of a homogeneous and relatively thick P3HT film, while CB enabled the (3BS)_2_-SiPc to swell into the P3HT layer. It was essential to deposit both layers dynamically to prevent the complete dissolution of the P3HT layer during deposition of (3BS)_2_-SiPc.

**Figure 2:**
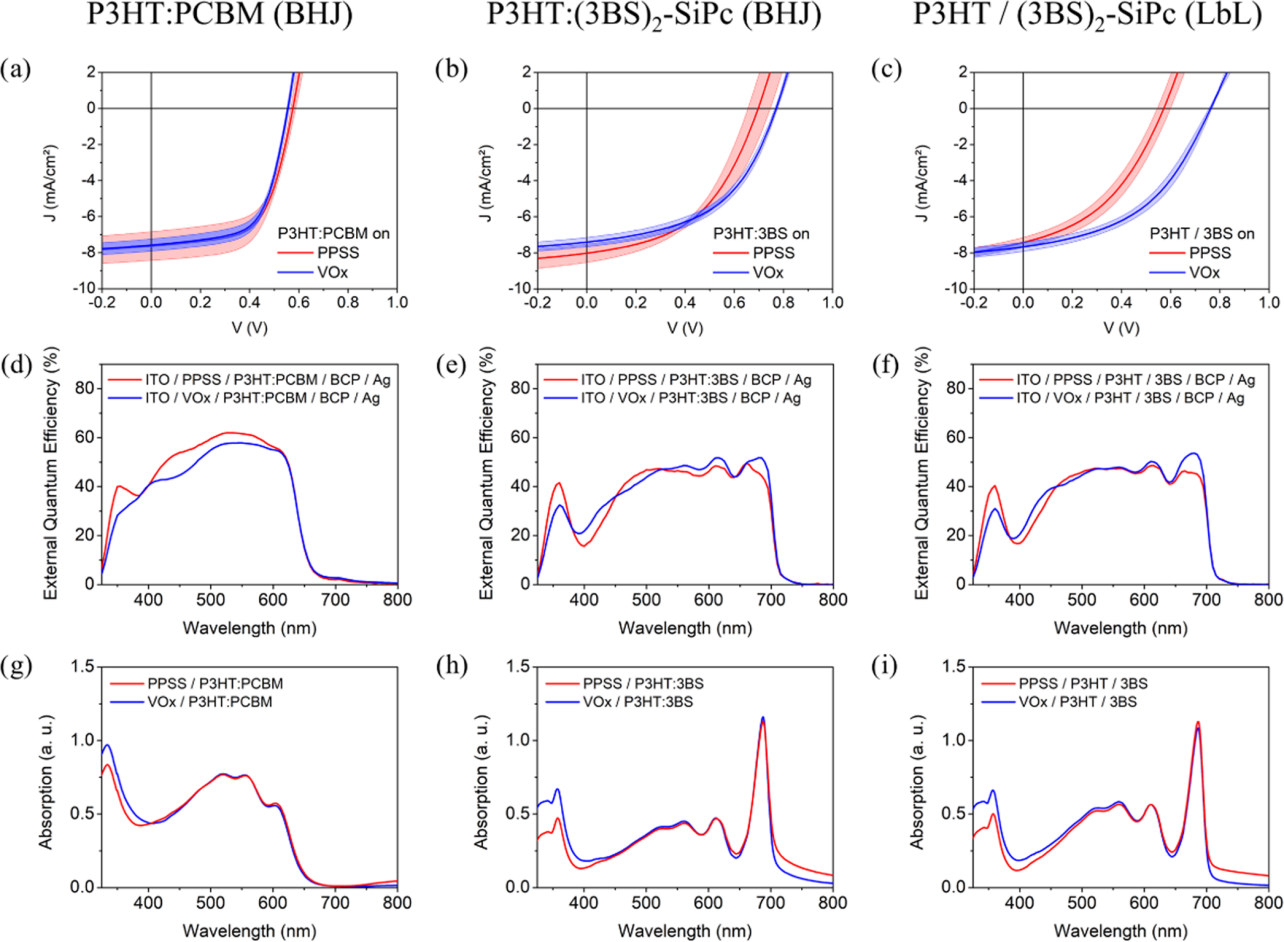
(a–c) Current vs voltage (*J*–*V*) curves with lines indicating the averaged curve and shades indicating the standard deviations, (d–f) external quantum efficiency (EQE) spectra, and (g–i) UV–vis absorption spectra for P3HT:PCBM BHJ, P3HT:(3BS)_2_-SiPc BHJ, and P3HT/(3BS)_2_-SiPc LbL on PEDOT:PSS (red) or VOx (blue) HTL. For convenience, (3BS)_2_-SiPc is referred to as 3BS and PEDOT:PSS as PPSS.

Current density–voltage (*J*–*V*) curves under 1 sun illumination for all devices using P3HT as the donor polymer and either PEDOT:PSS or VOx as the HTL are shown in Figure 2a–c, with corresponding electrical parameters summarized in Table 1. P3HT:PC_61_BM BHJ devices displayed very similar *V*_oc_, short-circuit current density (*J*_sc_), fill factor (FF), and PCE regardless of choice of HTL, which is consistent with previous reports comparing VOx to different HTLs, including PEDOT:PSS.^54,61−64^ For devices based on PEDOTS:PSS and VOx, an average PCE of 2.7 ± 0.3 and 2.6 ± 0.1%, with an averaged *V*_oc_ of 0.58 ± 0.01 and 0.56 ± 0.01 V, an averaged *J*_sc_ of 7.7 ± 0.8 and 7.6 ± 0.3 mA/cm^2^, and an averaged FF of 0.62 ± 0.02 and 0.63 ± 0.01 were obtained, respectively (for *n* = 14 devices). It is worth noting that the use of VOx seems to improve reproducibility in the baseline devices, as demonstrated by a drop in standard deviation (Table 1) and tightening in the spread of *J*–*V* curves (Figure 2a).

**Table 1:** *J*–*V* Characteristics for P3HT and (3BS)_2_-SiPc Integrated into Bulk and Bilayer Heterojunction Organic Photovoltaic Devices (0.325 cm^2^) with PEDOT:PSS (Abbreviated as PPSS) or VOx HTL^a^

		***I*–*V* parameters**		average ± SD [max]	
**HTL**	**active layer**	*V*_oc_ (V)	*J*_sc_ (mA/cm^2^)	FF	PCE (%)
PPSS	P3HT:PCBM *BHJ*	0.58 ± 0.01 [0.60]	7.7 ± 0.8 [8.8]	0.62 ± 0.02 [0.64]	2.7 ± 0.3 [3.1]
VOx		0.56 ± 0.01 [0.56]	7.6 ± 0.3 [8.1]	0.63 ± 0.01 [0.65]	2.6 ± 0.1 [2.8]
PPSS	P3HT:3BS *BHJ*	0.70 ± 0.05 [0.77]	8.0 ± 0.5 [8.6]	0.48 ± 0.02 [0.52]	2.7 ± 0.2 [3.0]
VOx		0.77 ± 0.01 [0.78]	7.4 ± 0.3 [7.9]	0.50 ± 0.02 [0.52]	2.8 ± 0.1 [3.0]
PPSS	P3HT/3BS *LbL*	0.57 ± 0.03 [0.61]	7.4 ± 0.3 [7.9]	0.41 ± 0.02 [0.45]	1.8 ± 0.2 [2.1]
VOx		0.76 ± 0.01 [0.77]	7.7 ± 0.3 [7.4]	0.46 ± 0.02 [0.49]	2.7 ± 0.2 [3.0]

a At least 14 devices were taken into consideration for the averages’ calculation.

Incorporation of (3BS)_2_-SiPc as the acceptor in BHJ devices with either HTL led to similar PCE performances compared to the baseline devices with a PC_61_BM acceptor, which represents a significant improvement over initial reports using (3BS)_2_-SiPc as an NFA in direct BHJ device configurations.^41^ While our PC_61_BM-based devices consistently achieved more favorable FF, the use of (3BS)_2_-SiPc resulted in higher *V*_oc_ (Table 1). Unlike PC_61_BM-based BHJ devices, the choice of HTL did impact *V*_oc_ in (3BS)_2_-SiPc-based devices, with an additional improvement from 0.70 V for PEDOT:PSS-based devices to 0.77 V for VOx-based devices, along with a slight improvement of the FF from 0.48 to 0.50, and improved consistency in performance. The increased *V*_oc_, 0.58 to 0.70 V going from one acceptor to the other, is due to the increased energy gap between the (3BS)_2_-SiPc LUMO and the P3HT HOMO (Figure 1), while the drop in FF, 0.62 to 0.48, is likely attributed to the reduced electron mobility of (3BS)_2_-SiPc compared to PC_61_BM and unfavorable morphology.

Similar trends in improved *V*_oc_ and HTL dependency were also observed in P3HT/(3BS)_2_-SiPc LbL devices (Figure 2c). In general, (3BS)_2_-SiPc LbL devices performed on par to their BHJ counterparts. When deposited on VOx, P3HT/(3BS)_2_-SiPc LbL devices had an enhanced PCE of 2.7 ± 0.2%, compared to 1.8 ± 0.2% for PEDOT:PSS, due to an increase in *V*_oc_ from 0.57 to 0.76 V and FF from 0.41 to 0.46 (Table 1). This increase in *V*_oc_ when using VOx instead of PEDOT:PSS could arise from a reduction of the injection barrier between the P3HT donor and the HTL. In literature, the work function of VOx is reported to range between −5.1 and −5.6 eV,^55^ compared to that of PEDOT:PSS with a work function of −5.2 eV.^54,62^ Moreover, PEDOT:PSS being a polymer provides a smoother surface compared to a metal oxide such as VOx that is rougher. This difference in the interface could impact how P3HT forms and crystallizes and how charges are collected and recombine. Analogous to BHJ results, the use of VOx resulted in more consistent (3BS)_2_-SiPc-based LbL devices (Figure 2c and Table 1), suggesting that for P3HT devices, a VOx HTL layer can result in superior OPV performances compared to PEDOT:PSS-based devices. Furthermore, our optimized results demonstrate that (3BS)_2_-SiPc is a viable alternative to PC_61_BM in P3HT-based OPVs, capable of producing devices with comparable performances in either BHJ or LbL architectures.

**Figure 3:**
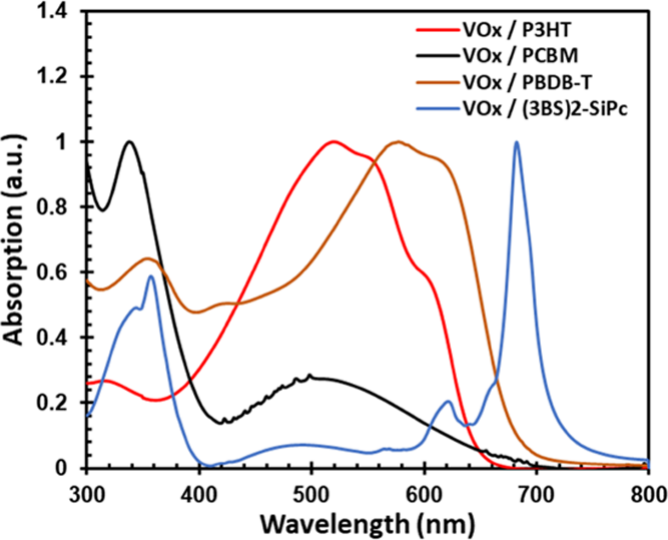
UV–vis absorption spectra for P3HT, PCBM, PBDB-T, and (3BS)_2_-SiPc films deposited on VOx.

To gain further insight into the relative contributions of the materials to photocurrent generation, external quantum efficiency (EQE) and UV–vis absorption measurements were conducted. UV–vis absorption measurements of individual materials are available in Figure 3. Figure 2d shows the EQE spectra of P3HT:PC_61_BM BHJ devices deposited on either PEDOT:PSS (red curve) or VOx (blue curve), which appear to be relatively similar in accordance with the *J*–*V* results. The EQE maximums around 525 nm for samples made on PEDOT:PSS or VOx are slightly above or below 60%, respectively, with a more prominent absorption peak for PEDOT:PSS around 340 nm and a dramatic decrease in absorption above 650 nm for both HTLs. The corresponding UV–vis absorption spectra (Figure 2g) are almost indistinguishable for the two HTLs, in accordance with the EQE trends.

Figure 2e shows the EQE spectra of P3HT:(3BS)_2_-SiPc BHJ devices deposited on either PEDOT:PSS or VOx. A more intense absorption peak is observed around 365 nm for samples made using PEDOT:PSS, while for films deposited on VOx, there is a more intense absorption peak around 680 nm and an absorption shoulder between 400 and 450 nm. In comparison to P3HT:PC_61_BM EQEs, the spectra have lower maxima around 50%, but the global absorptions are extended to after 700 nm thanks to the (3BS)_2_-SiPc contribution between 600 and 700 nm. This is confirmed with the corresponding UV–vis absorption spectra (Figure 2h), where the global absorption intensity before 650 nm dropped in comparison to P3HT:PC_61_BM, but a new highly intense absorption peak around 680 nm is observed for (3BS)_2_-SiPc.

EQE spectra for LbL devices of P3HT/(3BS)_2_-SiPc deposited on either PEDOT:PSS or VOx (Figure 2f) exhibit very similar trends compared to the BHJ devices. The same extension of the absorption range by approximately 70 nm compared to PC_61_BM-based devices is observed. The EQE maxima are still around 50%, with only a slight increase for devices made on VOx around 680 nm. Corresponding UV–vis spectra shown in Figure 2i for both HTLs are very similar to the blended active layers. These results further confirm that LbL fabrication imparts equivalent active layer optical properties compared to the BHJ approach and that (3BS)_2_-SiPc can enable an extension of the absorption range. Moreover, the choice of the HTL seems to only have little impact on the active layer absorption.

**Figure 4:**
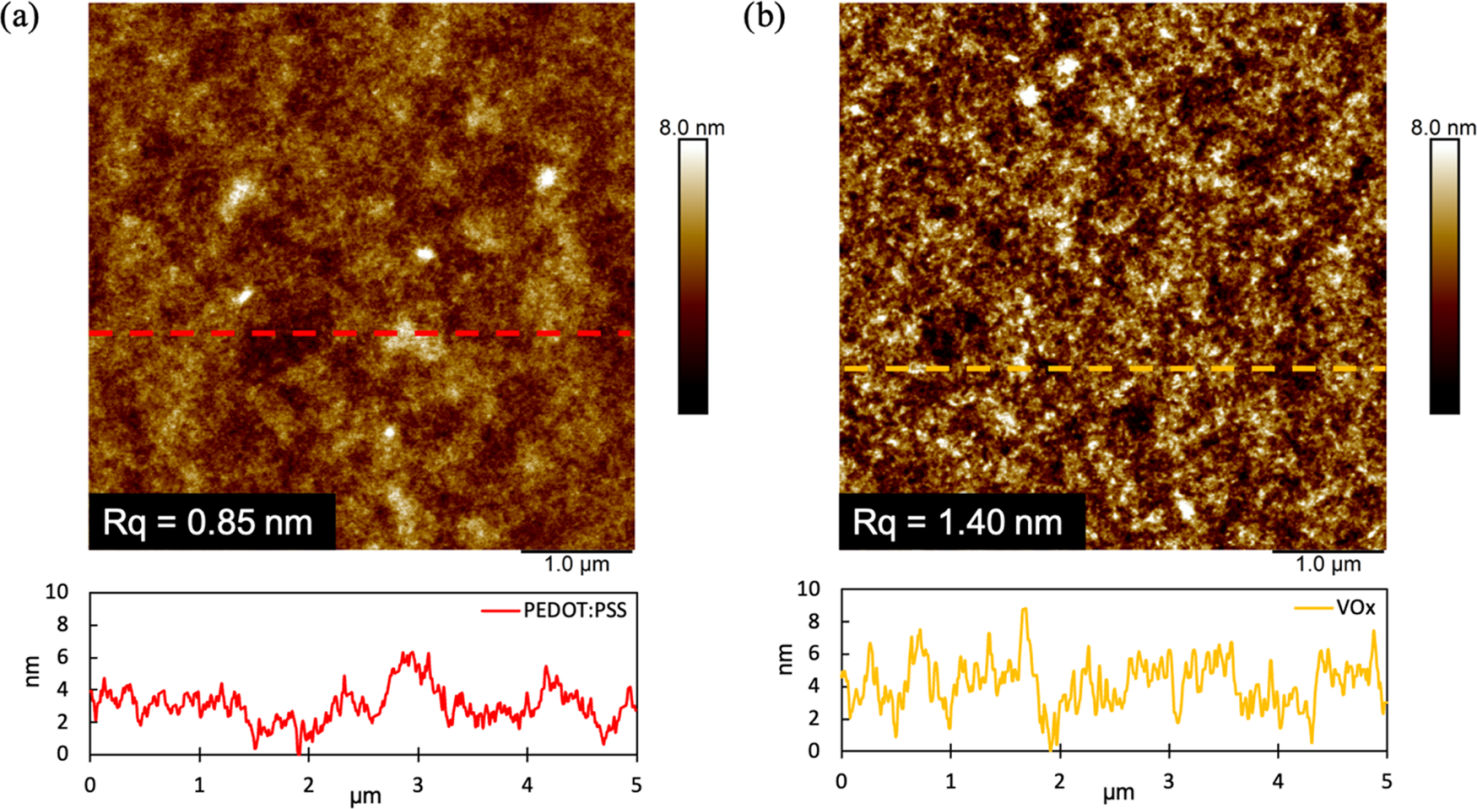
AFM height images of P3HT layers deposited onto (a) PEDOT:PSS and (b) VOx. The dashed lines indicate the location of the line segment height analysis, shown below the two images.

We surmised that the nature of the HTL layer provides different templating effects on the formation of the P3HT layer during deposition, which could influence the P3HT and (3BS)_2_-SiPc interface, ultimately leading to changes in device performances. Contact angle measurements did not reveal wettability differences between the two HTLs, with similar hydrophilic behaviors and angle values around 10° to water, and angle values around 4° to chloroform (solvent used for the P3HT layer) (Table S3). Atomic force microscopy (AFM) was used to characterize P3HT films (no acceptor molecules) deposited under identical conditions, either on PEDOT:PSS or VOx, with representative images shown in Figure 4. The P3HT film deposited on PEDOT:PSS (Figure 4a) was relatively smooth, with fairly consistent morphology and features, resulting in a low root-mean-square (RMS) roughness of 0.85 nm. In contrast, P3HT deposited on VOx (Figure 4b) is characterized by more pronounced peak-to-valley height differences in the line height section, with a dramatically larger RMS roughness of 1.40 nm, confirming that the choice of HTL does influence the morphology of the donor layer. From these findings, we assume that the VOx layer facilitates a more favorable templating surface for (3BS)_2_-SiPc onto P3HT, encouraging deeper interpenetration of the donor and acceptor at the interface, and potentially leading to increased donor/acceptor interfacial area and reduced charge recombination. This is consistent with the increased FF and *V*_oc_ of LbL VOx/P3HT/(3BS)_2_-SiPc devices discussed previously.

**Figure 5:**
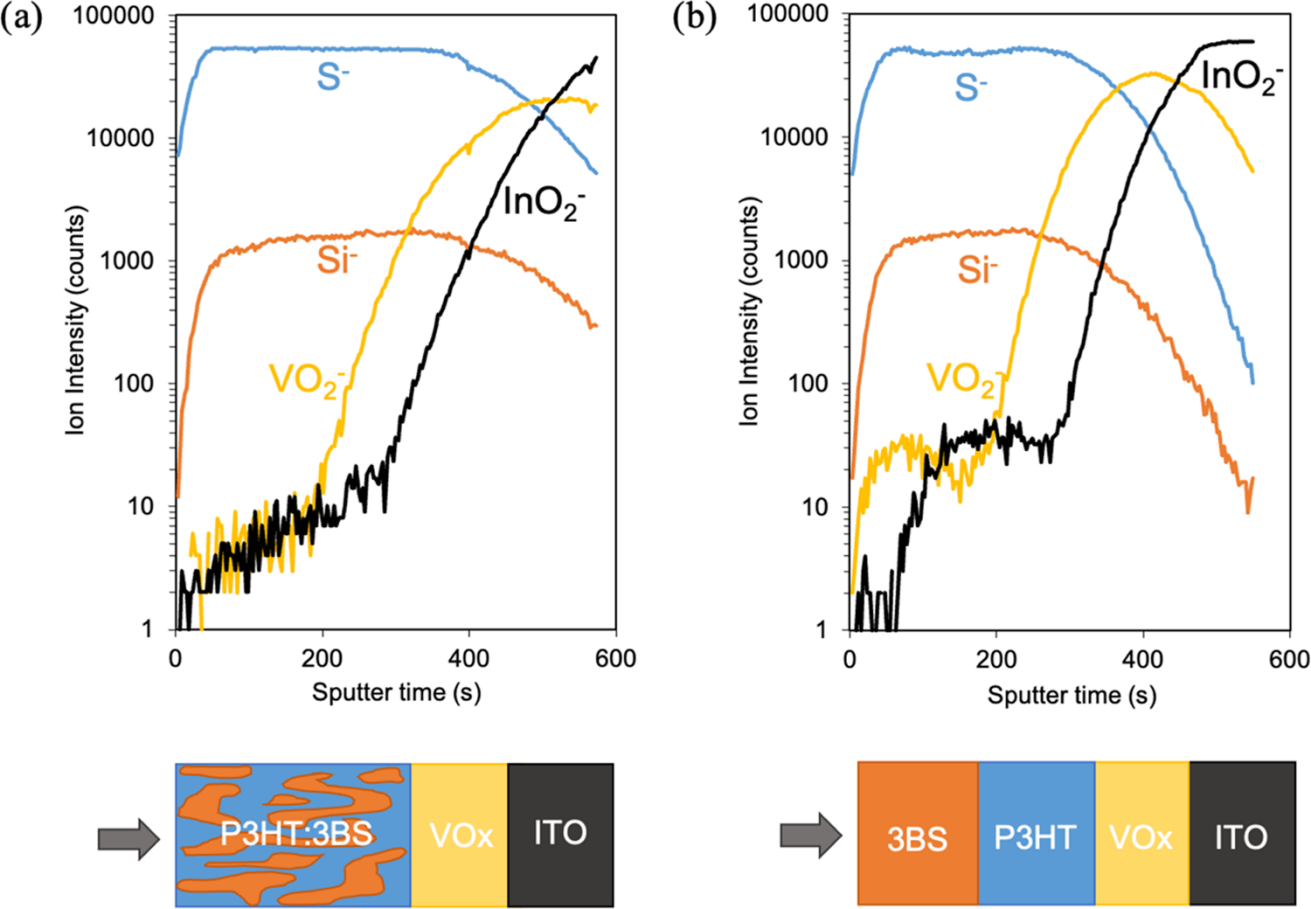
TOF-SIMS depth profiles of (a) blend VOx/P3HT:(3BS)_2_-SiPc and (b) LbL VOx/P3HT/(3BS)_2_-SiPc photoactive layers.

We conducted time-of-flight secondary ion mass spectroscopy (TOF-SIMS) measurements to elucidate the vertical concentration gradient profiles of P3HT and (3BS)_2_-SiPc through the entire thickness of both a BHJ (Figure 5a) and LbL (Figure 5b) active layer deposited on a VOx HTL. Sulfur ion (S^–^) was used to track P3HT, silicon ion (Si^–^) used for the (3BS)_2_^–^SiPc acceptor, vanadium oxide ion (VO_2_^–^) for the VOx HTL layer, and indium oxide ion (InO_2_^–^) was used to track the ITO electrode. No significant differences were noticed between the two architectures; the intensity profiles of S^–^ and Si^–^ signals are similar and homogeneous throughout the whole layer, suggesting that P3HT and (3BS)_2_-SiPc are evenly distributed throughout the films. For the BHJ structure, the sputter time is longer, indicating that it takes more time to go through the active layer compared to the LbL structure, which is consistent with the relative active layer thickness of the BHJ versus LbL devices obtained by profilometry. TOF-SIMS results for the LbL devices suggest a complete dissolution and intermixing of the (3BS)_2_-SiPc layer into the P3HT layer despite their sequential processing. We surmise the deposition of (3BS)_2_-SiPc induces a resolubilization of the P3HT layer despite the solvents’ immiscibility. When measuring film thicknesses, we observe a decrease from 160 nm for the neat P3HT layer to 110 nm for the P3HT/(3BS)_2_-SiPc bilayer. Moreover, dynamic spin coating of (3BS)_2_-SiPc led to visible discoloration of the P3HT layer that we assume is the (3BS)_2_-SiPc solution swelling and partially washing away the P3HT layer enabling the migration of (3BS)_2_-SiPc to the bottom of the film. A similar absence of a vertical separation for LbL devices has been reported for other systems, even when using immiscible solvents.^26,65,66^ The equivalent vertical phase separation obtained by BHJ and LbL processing of the active layer confirms that LbL processing results in analogous film morphologies and ultimately similar device performances compared to the conventional BHJ processing while being more suitable for eventual module commercialization.

**Figure 6:**
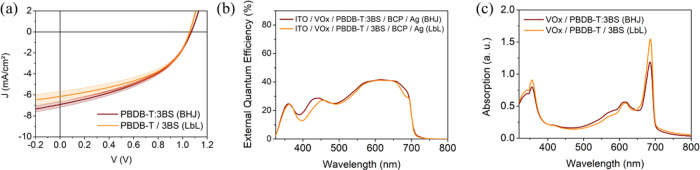
(a) Current vs voltage (*J*–*V*) curves with lines indicating the averaged curve and shades indicating standard deviations, (b) external quantum efficiency (EQE) spectra, and (c) UV–vis absorption spectra for PBDB-T:(3BS)_2_-SiPc BHJ (dark red) and PBDB-T/(3BS)_2_-SiPc LbL (orange) on VOx HTL. Both active layers were annealed at 100 °C for 10 min. For convenience, (3BS)_2_-SiPc is referred to as 3BS.

To assess the universality of our findings incorporating (3BS)_2_-SiPc into OPVs through the LbL approach, we combined our acceptor with another donor polymer. PBDB-T was chosen as PBDB-T/(3BS)_2_-SiPc has been demonstrated in an indirect BHJ device configuration to provide OPVs with PCE > 3.0% with *V*_oc_ > 1.0 V.^44^ PBDB-T:(3BS)_2_-Si BHJ devices and PBDB-T/(3BS)_2_-Si LbL devices were prepared using VOx as the HTL layer. The LbL process necessitated reoptimization of experimental conditions, resulting in slight changes in device fabrication conditions from P3HT to PBDB-T (described in detail in the Experimental Details and Supporting Information, Table S4). *J–V* curves, EQE spectra, and UV–vis absorption spectra for both LbL and BHJ device architectures are shown in Figure 6a–c, respectively, with electrical parameters summarized in Table 2. As with our P3HT system, BHJ devices and LbL devices using PBDB-T as the donor polymer had very similar device performances. PBDB-T:(3BS)_2_-SiPc BHJ devices achieved a PCE of 3.19 ± 0.13%, with a *V*_oc_ of 1.07 V, while the LbL devices had an average PCE of 3.02 ± 0.02%, with a *V*_oc_ of 1.06 V. These results are comparable to the performances obtained in the literature with a similar system using an indirect architecture.^44^ Even though BHJ devices attained a slightly improved average PCE compared to LbL devices, both devices displayed an impressive *V*_oc_ above 1 V due to the favorable frontier orbital offsets. These values are among the highest *V*_oc_ obtained for all-solution-processed LbL devices.^14^ As expected, the EQE spectra for both films (Figure 6b) are comparable, with a maximum slightly above 40%, and extended spectra up to 700 nm from the (3BS)_2_-SiPc contribution. Compared to the P3HT/(3BS)_2_-SiPc LbL system, the (3BS)_2_-SiPc contribution is decreased due to the similarities in band gaps between the two materials (Figure 1c). UV–vis absorption spectra for the two donor–acceptor films (Figure 6c) have matching trends, with an intense absorption peak just before 700 nm. These results demonstrate that (3BS)_2_-SiPc is an extremely versatile NFA for LbL processing, capable of replicating BHJ performances in different polymer systems.

**Table 2:** *J*–*V* Characteristics for PBDB-T and (3BS)_2_-SiPc Integrated into Bulk and Bilayer Heterojunction Organic Photovoltaic Devices (0.325 cm^2^) with VOx HTL^a^

	***I*–*V* parameters**		average ± SD [max]	
active layer annealed at 100 °C for 10 min	*V*_oc_ (V)	*J*_sc_ (mA/cm^2^)	FF	PCE (%)
PBDB-T:3BS *BHJ*	1.1 ± 0.01 [1.1]	6.9 ± 0.3 [7.4]	0.43 ± 0.01 [0.44]	3.2 ± 0.1 [3.4]
PBDB-T/3BS *LbL*	1.1 ± 0.0 [1.1]	6.2 ± 0.5 [6.8]	0.46 ± 0.01 [0.47]	3.0 ± 0.2 [3.3]

a At least 10 devices were taken into consideration for the averages’ calculation.

## Conclusions

In this study, we investigated the use of the synthetically facile phthalocyanine derivative (3BS)_2_-SiPc as an NFA in sequentially all-solution-processed LbL OPV devices. Two HTLs, PEDOT:PSS and VOx, were investigated as HTLs with VOx found to facilitate favorable changes in the P3HT film morphology, which resulted in improved FF and *V*_oc_ for (3BS)_2_-SiPc-based devices processed by LbL. After optimization, direct LbL devices fabricated with VOx achieved PCEs up to 3.0% when integrating P3HT as the donor polymer, and PCEs up to 3.3% for devices with PBDB-T as the donor polymer, with an impressive *V*_oc_ up to 1.06 V. These represent the greatest reported *PCE* for SiPc-based LbL OPV devices, with the *V*_oc_ value above 1 V among the highest achieved for both LbL and PBDB-T-based devices. When compared to their BHJ counterparts, LbL devices demonstrated equivalent efficiencies, with analogous EQE responses and absorption spectra, and commensurate vertical film composition. These results substantiate the promise of inexpensive and synthetically facile SiPc-based derivatives as NFAs that can be incorporated into LbL devices fabricated through a more scalable and roll-to-roll transferable method, demonstrating the significant potential for commercially viable OPV modules.

## Experimental Details

For the full Experimental section including materials, general device fabrication, and details on characterization, see the Supporting Information.

### LbL Active Layer

The following formulations are for optimal conditions; however, all parameters were optimized for each device structure and can be found in the Supporting Information. P3HT (15 mg/mL) or PBDB-T (12 mg/mL) was dissolved in chloroform (≥99%) and stirred for 4 h. P3HT (150 μL) was deposited by dynamic spin coating at 1000 rpm for 80 s (Spincoat G3P from Specialty Coating Systems), while PBDB-T (300 μL) was deposited via static spin coating using the same speed and time. (3BS)_2_-SiPc was dissolved in chlorobenzene (99.8%) at a concentration of either 15 mg/mL (for P3HT devices) or 12 mg/mL (for PBDB-T devices) and stirred overnight at 50 °C. (3BS)_2_-SiPc was deposited by dynamic spin coating (40 μL) at 3500 rpm for 60 s onto P3HT or by static spin coating (300 μL) onto PBDB-T; PBDB-T/(3BS)_2_-SiPc layers were then annealed in a nitrogen atmosphere at 100 °C for 10 min. The combined LbL active layer thicknesses were approximately 110 and 90 nm for P3HT/(3BS)_2_-SiPc and PBDB-T/(3BS)_2_-SiPc layers, respectively.

## Supporting Information Available

Detailed experimental section, full list optimization for devices’ conditions and resulting device performance, and contact angle measurements (PDF)
