# Supplementary material for: Layer-by-Layer Organic Photovoltaic Solar Cells Using a Solution-Processed Silicon Phthalocyanine Non-Fullerene Acceptor
Source: ACS Omega. 2022 Feb 22;7(9):7541–9. doi: 10.1021/acsomega.1c05715 (PMC8908506; doi:10.1021/acsomega.1c05715)
Supplement: Supplementary file 1 — ao1c05715_si_001.pdf [file ao1c05715_si_001.pdf]

Supporting Information

Layer-By-Layer Organic Photovoltaic Solar Cells using a Solution-Processed Silicon Phthalocyanine Non-Fullerene Acceptor

Marie D. M. Faure[1], Chloé Dindault[1], Nicole A. Rice[1], Benoît H. Lessard[1,2]*

1. Department of Chemical and Biological Engineering, University of Ottawa, 161 Louis Pasteur, Ottawa, ON, Canada, K1N 6N5
2. School of Electrical Engineering and Computer Science, University of Ottawa, 800 King Edward Ave. Ottawa, ON, Canada K1N 6N5

*Corresponding Author. E-mail: benoit.lessard@uottawa.ca

Experimental details

Materials

PEDOT:PSS (Clevios HTL Solar, Batch#SCA388-47) was purchased from Heraeus, and vanadium(V) oxytriisopropoxide (VOx precursor, Lot#MKBK2918V) and 2-propanol (IPA, 99.9%) were purchased from Sigma Aldrich. P3HT (Lot#BLS26-24, RR ≥95%, M_w = 45 kDa) was purchased from Rieke Metals and PBDB-T (Batch#BM8-071, M_w = 107 kDa, M_n = 53 kDa) was acquired from Brilliant Matters. $PC_{61}BM$ (99.5%) was acquired from Nano-C and $(3BS)_2$-SiPc was synthetized in house according to literature[44] and purified using train sublimation. Bathocuproine (BCP, sublimed >99.5%) powder was purchased from Lumtec and Ag (99.99%) pellets from Angstrom Engineering. All compounds were used as received unless otherwise specified.

Devices were prepared on 25 mm x 25 mm (± 0.2 mm) 0.7 mm thick glass substrates coated (via sputtering) with 1450 ± 100 Å of ITO (15 ± 3 ohms/sq, transmission: 89% Avg. @ 550nm) that were purchased from Thin Film Devices Inc.

General Device Fabrication

Direct structure, bilayer devices with an active area of 0.325 cm^2 were fabricated with the following structure: glass/ITO/HTL/donor layer/acceptor layer/BCP/Ag. ITO-coated glass substrates were cleaned by successive 5-min sonication baths of soapy water (water + detergent), water, acetone, and finally methanol prior to an air plasma treatment for 15 min. The vanadium-based HTL solution was prepared under N_2 atmosphere by dissolution of VOx precursor in IPA with a volume ratio of 1:70 in the absence of heat or agitation. The solution was then spin coated at 10,000 rpm for 30 s in air (Spincoat G3P from Specialy Coating Systems) before letting the samples rest in air for at least 1 h, to yield an approximately 10 nm thick layer. The PEDOT:PSS solution was spin coated at 3000 rpm for 30 s on substrates before being annealed under air at 140 °C for 15 min, resulting

in a layer that was approximately 30 nm thick. Substrates were then transferred to a nitrogen glovebox.

After deposition of the active layers (see below), BCP (9 nm) and Ag (70 nm) were thermally evaporated in a vacuum chamber (base pressure < 2 x 10^{-6} Torr) using an Angstrom EvoVac through a custom shadow mask to yield 5 devices per substrate, with an active area of 0.325 cm^2 per device.

BHJ Active Layer

P3HT (20 mg/mL) and $PC_{61}BM$ were dissolved in 1,2-dichlorobenzene (99%) with a 1:1 mass ratio and stirred overnight at 50 °C before deposition by static spin-coating at 800 rpm for 90 s. P3HT (15 mg/mL) and $(3BS)_2$-SiPc were dissolved in 1,2-dichlorobenzene (99%) with a 1:1 mass ratio, stirred overnight at 50 °C, and deposited by static spin-coating at 2000 rpm for 80 s. PBDB-T (10 mg/mL) and $(3BS)_2$-SiPc were dissolved in 1,2-dichlorobenzene (99%) with a 1:1 mass ratio, stirred at 35°C overnight, and deposited by static spin-coating at 1000 rpm for 120 s before annealing at 100 °C for 10 min under a nitrogen atmosphere. Thicknesses of approximately 150 and 120 nm were obtained for P3HT:$(3BS)_2$-SiPc and PBDB-T:$(3BS)_2$-SiPc BHJ layers, respectively.

Device Characterization

Current density vs. voltage (J-V) characteristics were measured using an Abet Technologies Sunlite 11,002 solar simulator under a nitrogen atmosphere (Xenon arc lamp) with an Air Mass 1.5 Global filter calibrated to 1 sun (1000 W/m^2) using a silicon reference cell (Abet 15150). External quantum efficiency (EQE) was measured over a 325 – 900 nm wavelength range using a Newport Oriel CS130-QUANTX. Layer thickness was assessed using a Brucker Dektak XT Profilometer, and AFM height images were obtained with a Bruker Dimension Icon in ScanAsyst mode, using

ScanAsyst-Air probes and a scan rate of 0.8 Hz. Calculation of root-mean-square (RMS) roughness and image processing were performed using NanoScope Analysis v1.8. Depth profiles were obtained by time-of-flight secondary ion mass spectrometry (TOF-SIMS) using an ION-TOF (GmbH) TOF-SIMS IV equipped with a Bi cluster liquid metal ion source (25 keV Bi_3^+, pulsed at 10 kHz). A depth profile was obtained by repeating the cycle of alternatively sputtering the surface in an area of 250 μm × 250 μm with a 3 keV Cs^+ beam for 3 s, waiting 1 s, and collecting an ion mass spectrum using the Bi_3^+ primary ion beam at 128 × 128 pixels over an area of 128 μm × 128 μm within the sputtered area (1 shot per pixel). Contact angle measurements were performed from three-point curve fitting using a VCA Optima goniometer (AST Products Inc) where droplets of DI water or chloroform were deposited by syringe onto glass/ITO/HTL substrates. UV-visible spectra of thin films were measured using an Agilent Cary 5000 UV-vis-NIR spectrophotometer in the 300 – 900 nm range.

Table S1: Optimization of experimental processing conditions for ITO/PEDOT:PSS/P3HT/(3BS)$_2$-SiPc/BCP/Ag LbL devices: *J-V* characteristics (best determined conditions are highlighted in bold and (3BS)$_2$-SiPc is referred to as 3BS). For spinning a / b / c: a is the rate in rpm, b is the dispensing kinetic (S for static, D for dynamic) and c is the volume in µL.

P3HT (15 mg/mL)			3BS (15 mg/mL)			I-V parameters			
Solvent	Spinning<br>a / b / c	Annealing	Solvent	Spinning<br>a / b / c	Annealing	V_{oc}<br>(V)	J_{sc}<br>(mA/cm^2)	FF	PCE<br>(%)
DCB	2000 / S / 200	-<br>10 min 80°C<br>20 min 80°C<br>30 min 80°C	DCB	2000 / S / 400	-	NaN	NaN	NaN	NaN
DCB	1000 / S / 200	-<br>20 min 80°C<br>-<br>20 min 80°C	DCB	3500 / S / var<br>3500 / S / var<br>3500 / D / var<br>3500 / D / var	-	NaN	NaN	NaN	NaN
CB	1000 / S / 200	-<br>-<br>20 min 80°C	CB	3500 / S / var<br>3500 / D / var<br>3500 / D / var	-	NaN<br>0.60 ± 0.07<br>0.61 ± 0.04	NaN<br>6.0 ± 0.5<br>6.4 ± 0.4	NaN<br>0.40 ± 0.07<br>0.43 ± 0.06	NaN<br>1.5 ± 0.5<br>1.7 ± 0.4
CB	1000 / S / 200	20 min 80°C	CB	1000 / D / var<br>2000 / D / var<br>2000 / D / var	-<br>-<br>10 min 150°C	0.52 ± 0.1<br>0.67 ± 0.06<br>0.68 ± 0.04	3.5 ± 2.0<br>5.6 ± 0.9<br>5.0 ± 0.3	0.33 ± 0.1<br>0.44 ± 0.08<br>0.46 ± 0.06	0.6 ± 0.4<br>1.7 ± 0.5<br>1.5 ± 0.2
**CF**	1000 / S / 200<br>1000 / S / 200<br>**1000 / D / 150**<br>1000 / D / 150	-<br>-<br>**-**<br>-	**CB**	3500 / S / 200<br>3500 / D / var<br>**3500 / D / 40**<br>3500 / D / var	-<br>-<br>**-**<br>10 min 100°C	0.48 ± 0.3<br>0.65 ± 0.02<br>**0.57 ± 0.03**<br>0.64 ± 0.03	4.0 ± 2.1<br>3.8 ± 0.8<br>**7.4 ± 0.3**<br>4.5 ± 1.3	0.34 ± 0.1<br>0.33 ± 0.01<br>**0.41 ± 0.02**<br>0.44 ± 0.05	1.0 ± 0.9<br>0.82 ± 0.2<br>**1.76 ± 0.19**<br>1.3 ± 0.5
CB	1000 / S / 200<br>1000 / S / 200<br>1000 / S / 200<br>1000 / D / 200<br>1000 / D / 200	-<br>-<br>20 min 80°C<br>-<br>20 min 80°C	TOL	3500 / S / 150<br>3500 / D / 150<br>3500 / D / 150<br>3500 / D / 150<br>3500 / D / 150	-	0.77 ± 0.01<br>0.71 ± 0.04<br>0.68 ± 0.07<br>0.67 ± 0.1<br>0.67 ± 0.05	6.1 ± 0.08<br>6.5 ± 0.5<br>5.1 ± 0.3<br>7.4 ± 0.2<br>6.7 ± 0.3	0.35 ± 0.01<br>0.33 ± 0.01<br>0.33 ± 0.01<br>0.37 ± 0.03<br>0.34 ± 0.01	1.7 ± 0.1<br>1.5 ± 0.2<br>1.1 ± 0.2<br>1.8 ± 0.4<br>1.6 ± 0.2
CF	1000 / S / 150	-	DCM:CB<br>100:0<br>60:40<br>20:80	3500 / D / 40	-	0.69 ± 0.05<br>0.75 ± 0.04<br>0.73 ± 0.09	0.46 ± 0.1<br>2.1 ± 0.2<br>4.5 ± 0.4	0.52 ± 0.06<br>0.33 ± 0.01<br>0.34 ± 0.02	0.17 ± 0.07<br>0.51 ± 0.04<br>1.1 ± 0.2
CF	1000 / S / 200<br>1000 / D / 150	-	CF	3500 / D / 40<br>3500 / D / 40	-	0.75 ± 0.01<br>0.64 ± 0.04	2.7 ± 0.3<br>6.9 ± 0.7	0.32 ± 0<br>0.39 ± 0.02	0.65 ± 0.07<br>1.7 ± 0.3

Table S2: Optimization of experimental processing conditions for ITO/VOx/P3HT/(3BS)$_2$-SiPc/BCP/Ag LbL devices: *J-V* characteristic (best determined conditions are highlighted in bold and (3BS)$_2$-SiPc is referred to as 3BS). For spinning a / b / c: a is the rate in rpm, b is the dispensing kinetic (S for static, D for dynamic) and c is the volume in μL.

P3HT (15 mg/mL)			3BS (15 mg/mL)			I-V parameters			
Solvent	Spinning a / b / c	Annealing	Solvent	Spinning a / b / c	Annealing	V_{oc} (V)	J_{sc} (mA/cm^2)	FF	PCE (%)
CF	1000 / S / 150	-	TOL	3500 / S / 150	-	0.76 ± 0.02	0.51 ± 0.50	0.34 ± 0.01	0.14 ± 0.14
	1000 / S / 150	20 min 80°C		3500 / S / 150		0.78 ± 0.01	1.16 ± 0.41	0.34 ± 0.01	0.31 ± 0.12
	1000 / S / 150	-		3500 / D / 150		0.75 ± 0.02	0.28 ± 0.04	0.30 ± 0.01	0.06 ± 0.01
	1000 / S / 150	20 min 80°C		3500 / D / 150		0.74 ± 0.02	0.21 ± 0.02	0.29 ± 0.00	0.05 ± 0.00
	1000 / D / 150	-		3500 / S / 150		0.77 ± 0.02	4.87 ± 0.30	0.36 ± 0.01	1.34 ± 0.08
	1000 / D / 150	20 min 80°C		3500 / S / 150		0.77 ± 0.01	4.49 ± 0.49	0.36 ± 0.01	1.24 ± 0.14
	1000 / D / 150	-		3500 / D / 150		0.80 ± 0.01	2.92 ± 0.18	0.35 ± 0.01	0.82 ± 0.05
	1000 / D / 150	20 min 80°C		3500 / D / 150		0.79 ± 0.03	2.10 ± 0.33	0.35 ± 0.02	0.58 ± 0.12
CB	1000 / S / 150	-	TOL	3500 / S / 150	-	0.79 ± 0	7.99 ± 0.14	0.41 ± 0.01	2.56 ± 0.10
	1000 / S / 150	20 min 80°C		3500 / S / 150		0.78 ± 0.01	6.02 ± 1.07	0.38 ± 0.01	1.77 ± 0.34
	1000 / S / 150	20 min 80°C		3500 / D / 150		0.78 ± 0.02	5.41 ± 0.49	0.37 ± 0.02	1.59 ± 0.23
	1000 / D / 150	-		3500 / S / 150		0.75 ± 0.04	7.44 ± 0.45	0.40 ± 0.02	2.22 ± 0.18
	1000 / D / 150	20 min 80°C		3500 / S / 150		0.78 ± 0.01	7.63 ± 0.38	0.40 ± 0.01	2.40 ± 0.19
	1000 / D / 150	-		3500 / D / 150		0.79 ± 0.01	6.46 ± 0.47	0.39 ± 0	1.97 ± 0.15
	1000 / D / 150	20 min 80°C		3500 / D / 150		0.79 ± 0	6.57 ± 0.21	0.39 ± 0.01	2.03 ± 0.09
**CF**	1000 / S / 150	-	**CB**	3500 / S / 150	-	0.76 ± 0.02	6.18 ± 0.31	0.57 ± 0.01	2.64 ± 0.22
	1000 / S / 150	20 min 80°C		3500 / S / 40	-	0.79 ± 0.01	5.11 ± 0.88	0.41 ± 0.01	1.63 ± 0.27
	1000 / S / 150	-		3500 / D / 150	-	0.78 ± 0.01	4.21 ± 1.13	0.43 ± 0.05	1.42 ± 0.56
	1000 / S / 150	20 min 80°C		3500 / D / 40	-	0.79 ± 0	2.18 ± 0.32	0.35 ± 0	0.60 ± 0.08
	1000 / D / 150	-		3500 / S / 150	-	0.72 ± 0.02	1.64 ± 0.65	0.61 ± 0.03	0.73 ± 0.32
	1000 / D / 150	20 min 80°C		3500 / D / 20	-	0.77 ± 0.02	6.59 ± 1.10	0.48 ± 0.06	2.44 ± 0.58
	1000 / S / 150	-		3500 / D / 40	-	0.77 ± 0.01	2.53 ± 0.35	0.42 ± 0.01	0.83 ± 0.13
	1000 / S / 150	20 min 80°C		3500 / D / 40	-	0.78 ± 0.01	2.36 ± 0.59	0.40 ± 0.02	0.75 ± 0.21
	**1000 / D / 150**	**-**		**3500 / D / 40**	**-**	**0.76 ± 0.01**	**7.66 ± 0.25**	**0.46 ± 0.02**	**2.68 ± 0.15**
	1000 / D / 150	-		3500 / D / 40	10 min 150°C	0.76 ± 0.01	6.44 ± 0.19	0.54 ± 0.03	2.65 ± 0.08
	1000 / D / 150	40 min 80°C		3500 / D / 40	10 min 150°C	0.77 ± 0.01	5.76 ± 0.28	0.51 ± 0.03	2.27 ± 0.18
	1000 / D / 150	10 min 150°C		3500 / D / 40	10 min 150°C	0.76 ± 0.01	6.12 ± 0.18	0.56 ± 0.02	2.62 ± 0.05
CF	1000 / S / 150	-	DCM:CB 100:0	3500 / D / 40	-	NaN	NaN	NaN	NaN
			60:40			0.77 ± 0.01	0.99 ± 0.09	0.34 ± 0.01	0.26 ± 0.02
			20:80			0.77 ± 0	1.6 ± 0.45	0.35 ± 0.01	0.44 ± 0.13

Table S3: Contact angle values (in °) of water or chloroform on ITO/PEDOT:PSS and ITO/VOx stacks.

	Contact angle (°) (averaged over 3 measurements)	
	Water	Chloroform
VOx	8	4
PEDOT:PSS	11	4

Table S4: Optimization of experimental processing conditions for ITO/VOx/PBDB-T/(3BS)2-SiPc/BCP/Ag LbL devices: J-V characteristics (best determined conditions are highlighted in bold and $(3BS)_2$-SiPc is referred to as 3BS). For spinning a / b / c: a is the rate in rpm, b is the dispensing kinetic (S for static, D for dynamic) and c is the volume in µL.

PBDB-T			3BS			I-V parameters			
Solvent / Concentration (mg/mL)	Spinning a / b / c	Annealing	Solvent / Concentration (mg/mL)	Spinning a / b / c	Annealing	V_{oc} (V)	J_{sc} (mA/cm²)	FF	PCE (%)
CF / 10	1000 / S / 200<br>1000 / D / 200<br>1000 / S / 200	-	CB / 10	3500 / D / 200<br>3500 / D / 200<br>3500 / S / 200	-	0.98 ± 0.1<br>0.98 ± 0.05<br>1.03 ± 0.04	0.57 ± 0.5<br>5.1 ± 0.4<br>4.7 ± 0.6	0.29 ± 0.01<br>0.32 ± 0.01<br>0.44 ± 0.02	0.97 ± 0.1<br>1.6 ± 0.2<br>2.2 ± 0.3
**CF / 12**	1000 / S / 200<br>**1000 / S / 300**<br>1000 / S / 300	**-**	**CB / 12**	3500 / S / 200<br>**3500 / S / 300**<br>3500 / S / 300	-<br>**10 min 100°C**<br>10 min 150°C	1.07 ± 0.01<br>**1.06 ± 0.0**<br>1.04 ± 0.01	6.1 ± 0.2<br>**6.15 ± 0.48**<br>6.0 ± 0.05	0.42 ± 0.01<br>**0.46 ± 0.01**<br>0.47 ± 0.01	2.7 ± 0.09<br>**3.02 ± 0.20**<br>2.9 ± 0.05 [d]
CF / 12	1000 / S / 200<br>1000 / D / 200	-	CF / 12	3500 / S / 200<br>3500 / D / 200	-	1.05 ± 0.02<br>1.05 ± 0.01	3.0 ± 0.3<br>1.7 ± 0.2	0.36 ± 0.01<br>0.28 ± 0.00	1.1 ± 0.1<br>0.50 ± 0.06

[d] significant number of short-circuited devices (6 devices out of 10)
